# Resveratrol-mediated Fe(III)/peroxydisulfate activation for ofloxacin degradation at circumneutral pH

**DOI:** 10.1016/j.isci.2026.117733

**Published:** 2026-09-22

**Authors:** Yanlin Wu, Wenyu Yang, Yilan Zeng, Dingfeng Huang, Wenbo Dong, Olivier Monfort, Martin Motola

**Affiliations:** 1 School of Resources and Environmental Engineering, Shanghai Polytechnic University, Shanghai 201209, China; 2 Department of Inorganic Chemistry, Faculty of Natural Sciences, Comenius University Bratislava, Ilkovicova 6, 842 15 Bratislava, Slovakia; 3 Shanghai Key Laboratory of Air Quality and Environmental Health, Department of Environmental Science & Engineering, Fudan University, Shanghai 200433, China; 4 Centre for Nanotechnology and Advanced Materials, Faculty of Mathematics Physics and Informatics, Comenius University Bratislava, Mlynska dolina, 842 48 Bratislava, Slovakia

**Keywords:** Fe-redox mediation, natural reductant, ofloxacin degradation, PDS activation, resveratrol

## Abstract

Iron-mediated peroxydisulfate (PDS) activation is limited by slow Fe(III)/Fe(II) redox cycling under circumneutral conditions. Here, resveratrol (RES), a natural stilbene polyphenol, was introduced as a low-dose redox mediator to promote Fe(II) formation from Fe(III) for enhanced PDS activation. The Fe(III)/RES/PDS system showed higher ofloxacin (OFL) degradation than the corresponding single-reagent and binary systems and achieved approximately 90% OFL degradation at pH 6.5. Chemical quenching and competition-kinetic analysis indicated that sulfate radical (SO_4_^·−^) was the major contributor to OFL degradation, while hydroxyl radical (^·^OH) played a secondary role. Matrix experiments showed that Cl^−^ and NO_3_^−^ had minor effects, whereas NO_2_^−^ strongly inhibited OFL degradation. This work provides a case study of RES-mediated Fe(III)/PDS activation for antibiotic degradation under near-neutral conditions.

## Highlights

• Natural resveratrol can assist Fe(III)-mediated PDS activation
• RES promotes Fe(III) reduction and supports Fe(III)/Fe(II) cycling
• Balanced RES concentration improves redox cycling while limiting radical competition
• Near-neutral OFL degradation is achieved without additional pH adjustment

## Graphical abstract

**Figure:**
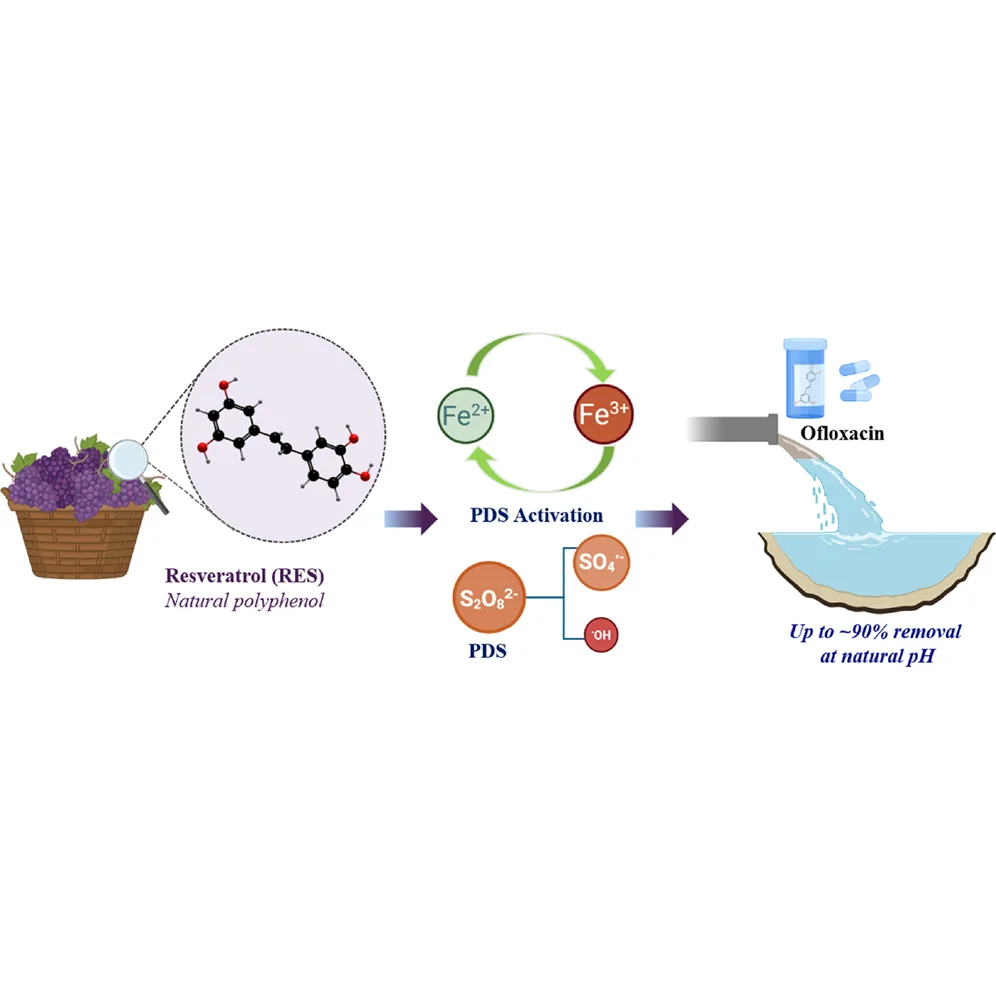

## Teaser

Catalysis; Environmental science; Environmental chemistry

## Introduction

Fluoroquinolone antibiotics are frequently detected in aquatic environments because of their extensive application in human medicine, livestock production, and aquaculture.^1^^,^^2^ Ofloxacin (OFL), as a representative fluoroquinolone (Figure 1A), is of particular concern due to its poor biodegradability, ecological toxicity, and potential contribution to the spread of antibiotic resistance.^3^ Conventional wastewater treatment processes are often insufficient for the complete removal of such persistent antibiotics, which has stimulated the research and development of advanced oxidation processes (AOPs) for their degradation in water.^4^^,^^5^

**Figure 1:**
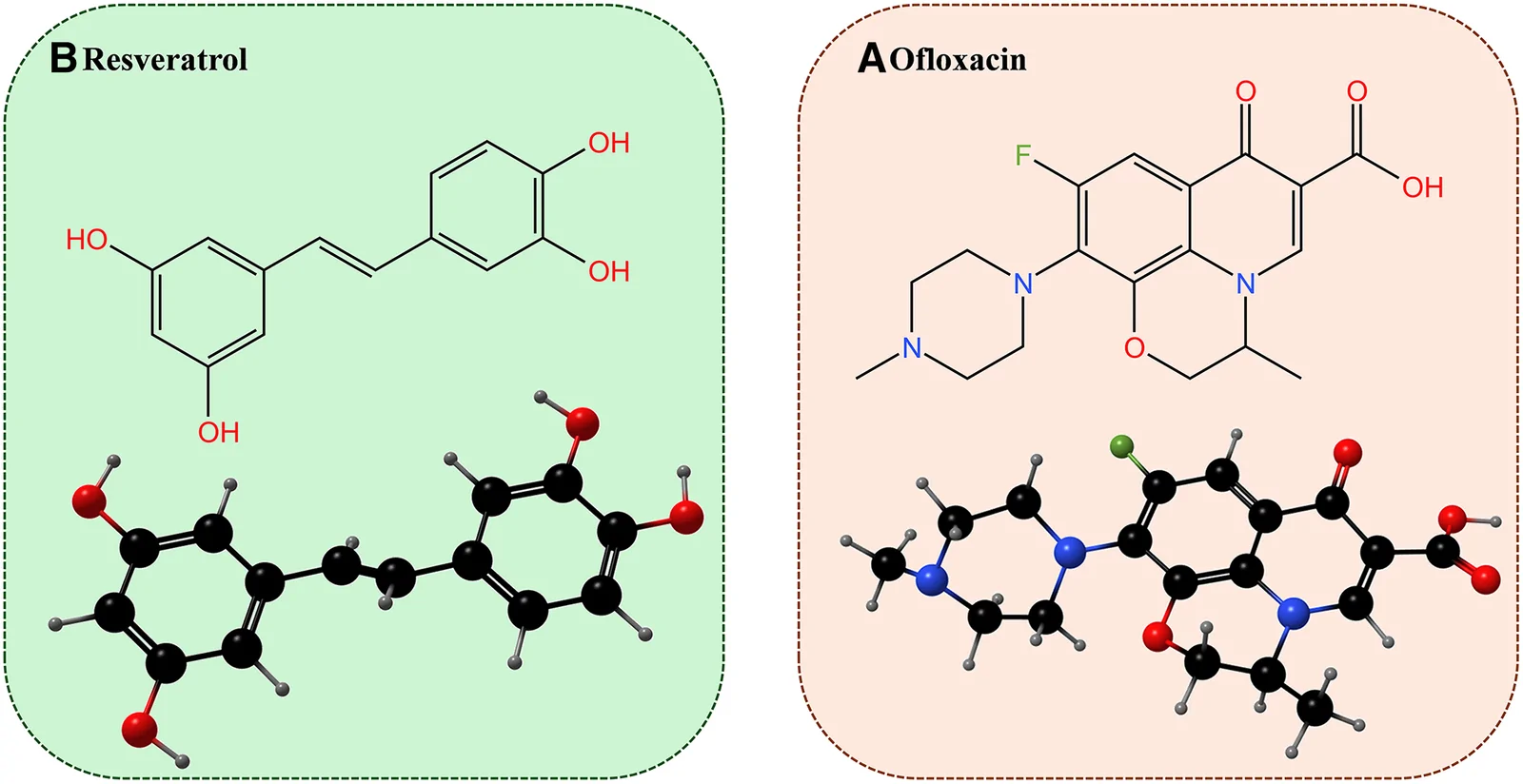
Chemical structures and optimized molecular configurations (A) Ofloxacin. (B) Resveratrol.

Among AOPs, sulfate radicals-based advanced oxidation process (SR-AOPs) has attracted increasing attention because peroxydisulfate (PDS) and peroxymonosulfate (PMS) can be activated to generate highly reactive species, especially sulfate radicals (SO_4_^·−^) and hydroxyl radicals (^·^OH).^5^^,^^6^^,^^7^ Compared with ^·^OH, SO_4_^·−^ generally has longer lifetime, higher redox potential, and better adaptability over a broader pH range.^7^^,^^8^ Transition metal-mediated PDS activation is particularly attractive as it can operate without external energy input and relies on simple reagent combinations.^9^^,^^10^ Iron species are among the most widely studied activators because of their low cost, environmental compatibility, and strong redox activity.^11^^,^^12^ However, iron-mediated PDS activation is limited by the slow Fe(III)/Fe(II) redox cycle.^13^^,^^14^ Under circumneutral and alkaline conditions, iron species tend to aggregate and precipitate into iron oxyhydroxide, thus further reducing the availability of active iron species and leading to inefficient PDS activation.^11^^,^^15^

Employing reductive mediators is an effective strategy to accelerate Fe(III) reduction and improve the utilization of iron in SR-AOPs.^13^^,^^16^ Natural polyphenols have attracted increasing attention nowadays owing to their abundant phenolic hydroxyl groups, which facilitate electron transfer and, in some cases, interactions with metal ions.^9^^,^^17^^,^^18^ In iron-based AOPs, polyphenols can promote Fe(II) regeneration, maintain iron in a more reactive state, and consequently enhance peroxide or persulfate activation.^9^^,^^18^^,^^19^^,^^20^ However, the effect of polyphenols is often concentration dependent.^21^^,^^22^^,^^23^ At elevated concentrations, naturally derived polyphenols may shift from redox mediators to radical competitors by scavenging reactive species or competing with target pollutants for oxidizing species reaction, thereby affecting overall degradation efficiency.^13^^,^^24^^,^^25^ This concentration-dependent behavior suggests that the function of polyphenols in iron-mediated persulfate systems should be considered a balance between redox promotion and reactive species consumption.^26^ Therefore, resveratrol (RES), a naturally occurring stilbene polyphenol widely distributed in plants (e.g., grapes and Japanese knotweed), was selected as a representative natural mediator for Fe(III)-based PDS activation (Figure 1B).^27^ Although polyphenol-assisted iron redox cycling has been widely reported in Fenton-like and persulfate-based AOPs, most previous studies have focused on phenolic acids, catechol-containing compounds, or highly reducing polyphenols, such as gallic acid, catechin, chlorogenic acid, protocatechuic acid, and tannic acid.^9^^,^^18^^,^^20^^,^^23^ Compared with these commonly reported mediators, RES has a stilbene backbone, a different phenolic hydroxyl distribution, and relatively low aqueous solubility, which may lead to different reducing behavior and a narrower effective mediation window.

In this study, an RES-mediated Fe(III)/PDS system was developed for OFL degradation under circumneutral conditions. The capability of RES to reduce Fe(III) to Fe(II) was examined to establish its role in iron redox mediation. The degradation performance of OFL was then evaluated and compared to that of single-component and binary systems. Radical quenching experiments were conducted to assess the contribution of reactive species, and the effects of common inorganic ions in aqueous matrices were assessed. This work aims to evaluate the performance of RES-assisted Fe(III)/PDS activation and to provide preliminary insight into its possible role in iron redox mediation.

## Results

### Initial evidence for RES-mediated Fe(III) reduction

Fe(II) formation was observed after RES was introduced into Fe(III) solutions, indicating that RES can reduce Fe(III) under the tested conditions (Table 1).^26^ At an initial Fe(III) concentration of 50 μM, the addition of 10 and 25 μM RES produced 3.075 and 10.00 μM Fe(II), corresponding to reduction efficiencies of 6.2% and 20.00%, respectively. When the Fe(III) concentration was increased to 200 μM, the reduction efficiencies decreased to 5.1% and 15.3% at the same RES concentrations. These results show that Fe(II) formation depended on the relative concentrations of RES and Fe(III).

**Table 1:** Fe(II) formation from Fe(III) reduction in the presence of RES without PDS

Composition	Fe(II) concentration (μM)	Reduction
50 μM Fe(III)+10 μM RES	3.075	6.15%
50 μM Fe(III)+25 μM RES	10.00	20.00%
200 μM Fe(III)+10 μM RES	10.16	5.08%
200 μM Fe(III)+25 μM RES	30.50	15.25%

### OFL degradation in different reaction systems

Fe(III), PDS, and RES alone, as well as the binary Fe(III)/RES and RES/PDS systems, all produced only limited OFL removal, while the binary Fe(III)/PDS system showed moderate degradation activity (Figures 2A and 2B). The higher OFL removal in the Fe(III)/RES/PDS system indicates that the presence of RES improved Fe(III)-based PDS activation.^27^

**Figure 2:**
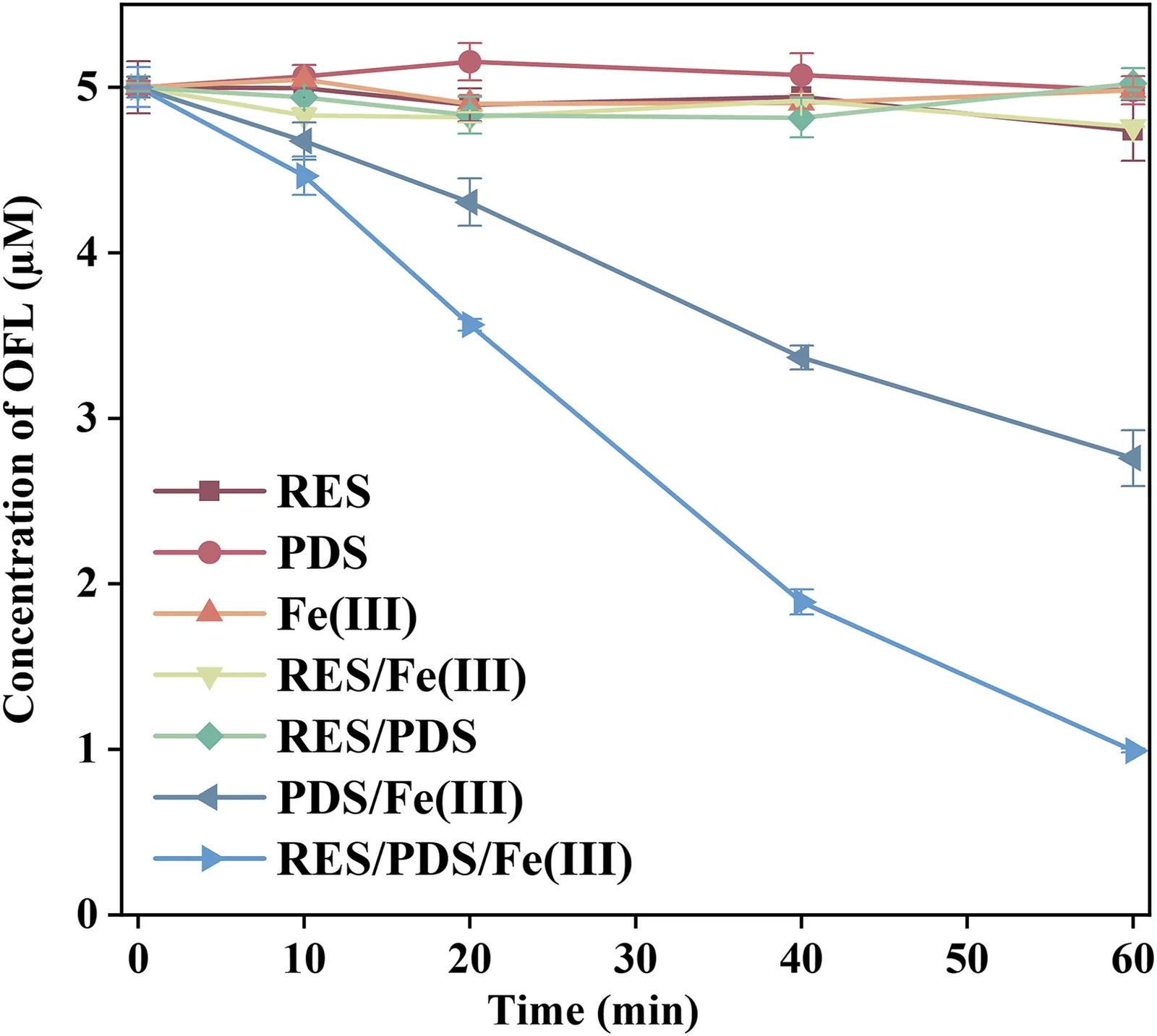
OFL degradation in different systems All experiments were conducted at [OFL]_0_ = 5 μM, [Fe(III)]_0_ = 100 μM, [PDS]_0_ = 500 μM, [RES]_0_ = 10 μM, and pH = 6.5. Error bars represent mean ± SD (*n* = 3).

### Effect of reagents’ concentration on OFL degradation

Increasing the PDS concentration from 250 to 500 μM improved OFL degradation (Figure 3A), as indicated by a faster decrease in OFL concentration and a higher *k*_*obs*_ (Figure 3B). This improvement indicates that the reaction benefited from a higher amount of activated PDS within this concentration range. However, when the PDS concentration was further increased to 750 μM, the additional enhancement was minor. Based on this trend, 500 μM PDS was used in the following experiments.

**Figure 3:**
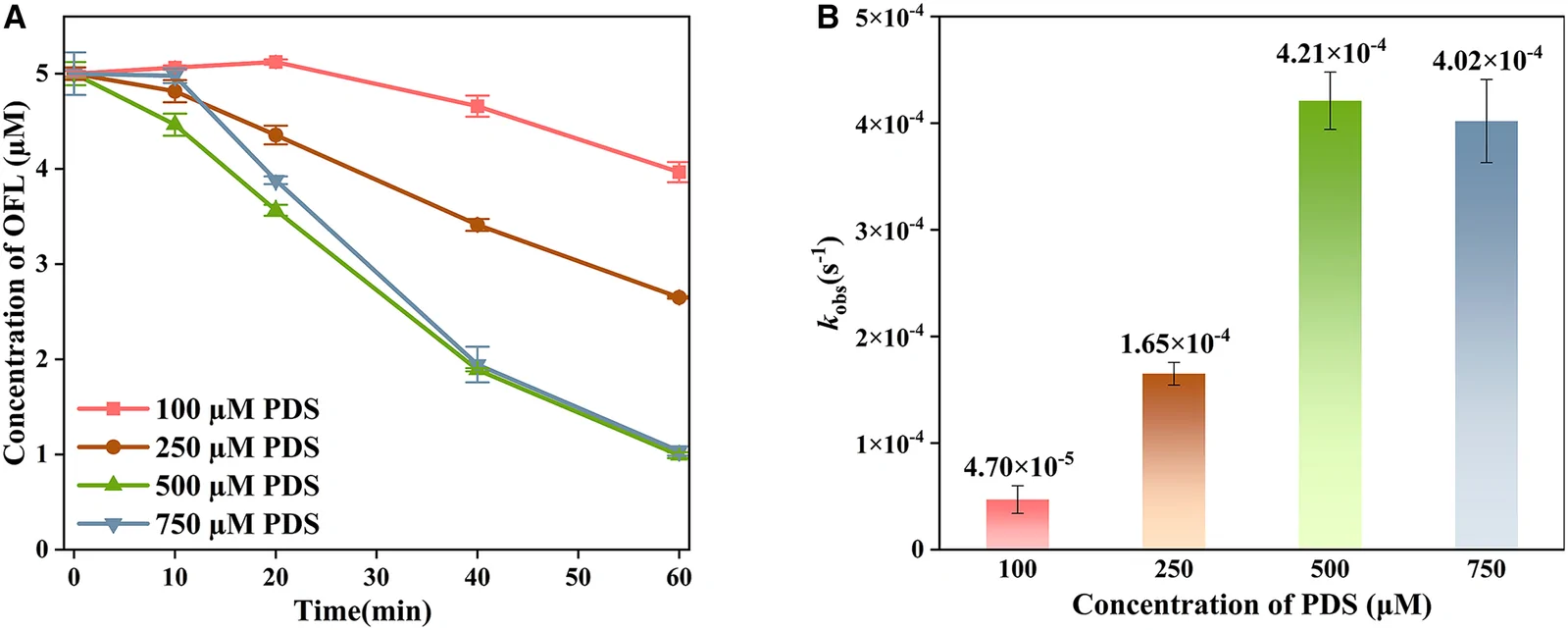
Effect of PDS concentration in the Fe(III)/RES/PDS system (A) OFL degradation. (B) Rate constants (*k*_*obs*_). All experiments were conducted at [OFL]_0_ = 5 μM, [Fe(III)]_0_ = 100 μM, [RES]_0_ = 10 μM, and pH = 6.5. Error bars represent mean ± SD (*n* = 3).

Fe(III) concentration also affected OFL degradation. Increasing the Fe(III) concentration from 50 to 300 μM accelerated the decrease in OFL concentration (Figure 4A), with the corresponding *k*_*obs*_ also increasing from 2.94 × 10^−4^ to 6.84 × 10^−4^ s^−1^ (Figure 4B). The most pronounced improvement occurred between 100 and 150 μM Fe(III), where *k*_*obs*_ increased from 3.98 × 10^−4^ to 5.86 × 10^−4^ s^−1^. Further increasing Fe(III) from 150 to 300 μM produced only a less significant increase in *k*_*obs*_. Although 300 μM Fe(III) gave the highest rate constant, 150 μM Fe(III) was selected for subsequent experiments as a balanced concentration that provided clear kinetic enhancement while avoiding excessive iron input.

**Figure 4:**
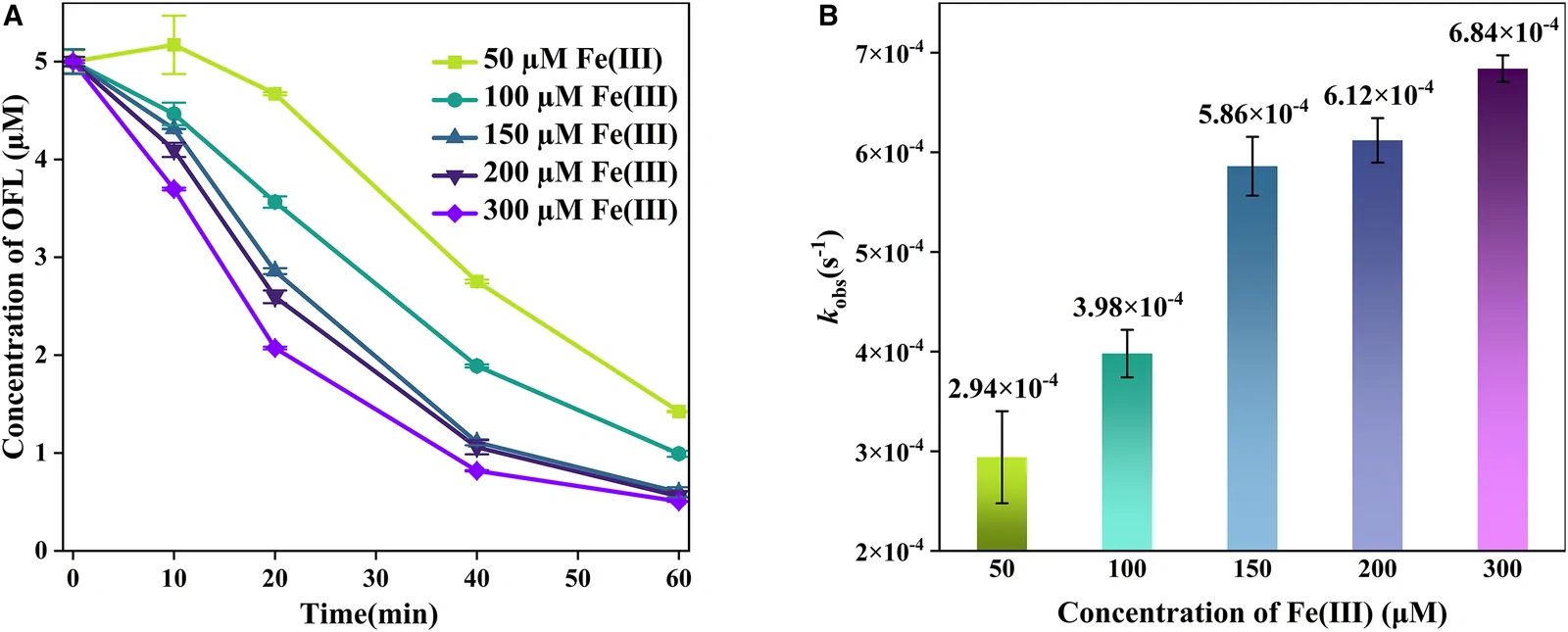
Effect of Fe(III) concentration in the Fe(III)/RES/PDS system (A) OFL degradation. (B) Rate constants (*k*_*obs*_). All experiments were conducted at [OFL]_0_ = 5 μM, [PDS]_0_ = 500 μM, [RES]_0_ = 10 μM, and pH = 6.5. Error bars represent mean ± SD (*n* = 3).

OFL degradation was also clearly enhanced when the RES concentration increased from 5 to 7.5 μM (Figure 5A). The corresponding *k*_*obs*_ increased from 3.19 × 10^−4^ to 5.51 × 10^−4^ s^−1^ (Figure 5B), suggesting the positive contribution of RES in promoting Fe(III)/PDS activation. A further increase in RES concentration to 10 μM resulted in a slightly higher *k*_*obs*_ of 5.86 × 10^−4^ s^−1^, while the performance at 15 μM was comparable but slightly lower. However, when the RES concentration was increased to 20 μM, OFL degradation decreased, with the *k*_*obs*_ decreasing to 3.53 × 10^−4^ s^−1^. Therefore, 10 μM RES was selected as the optimal concentration because it provided the highest rate constant under the tested conditions, whereas further increasing RES did not improve OFL degradation.

**Figure 5:**
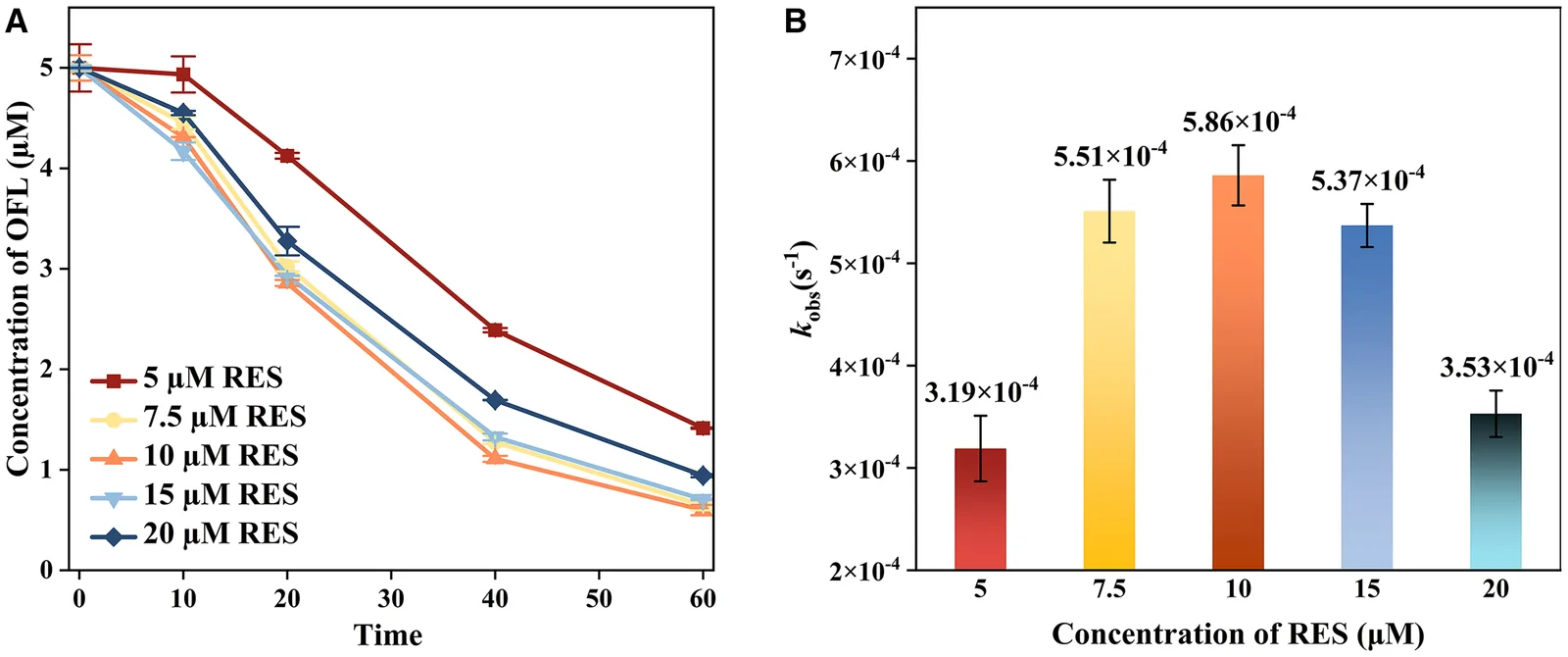
Effect of RES concentration in the Fe(III)/RES/PDS system (A) OFL degradation. (B) Rate constants (*k*_*obs*_). All experiments were conducted at [OFL]_0_ = 5 μM, [PDS]_0_ = 500 μM, [Fe(III)]_0_ = 150 μM, and pH = 6.5. Error bars represent mean ± SD (*n* = 3).

### Effect of pH on OFL degradation

The degradation of OFL was strongly dependent on the initial pH as demonstrated in Figure 6. The OFL degradation was negligible at pH 2.0 and 10.5, whereas it was relatively efficient under mildly acidic to circumneutral conditions (Figure 6A). The corresponding *k*_*obs*_ reached the maximum value of 5.86 × 10^−4^ s^−1^ at pH 6.5 (Figure 6B), suggesting circumneutral conditions as favorable pH for the Fe(III)/RES/PDS system.

**Figure 6:**
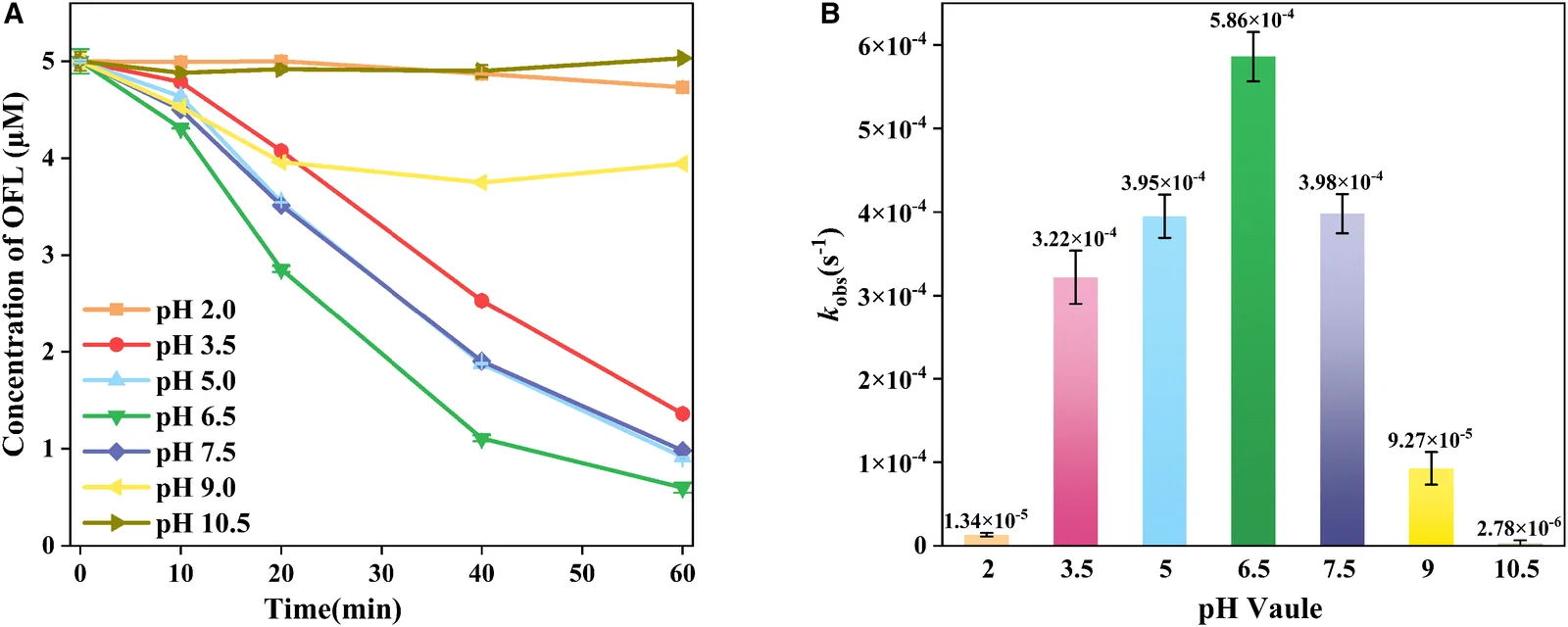
Effect of pH in the Fe(III)/RES/PDS system (A) OFL degradation. (B) Rate constants (*k*_*obs*_). All experiments were conducted at [OFL]_0_ = 5 μM, [Fe(III)]_0_ = 100 μM, [PDS]_0_ = 500 μM, and [RES]_0_ = 10 μM. Error bars represent mean ± SD (*n* = 3).

### Contribution of radical species in OFL degradation

As shown in Figure 7 and Table S1, the addition of 20 mM isopropyl alcohol (IPA) almost completely suppressed OFL degradation leaving a high residual OFL concentration after the reaction. Since IPA can react with both ^·^OH and SO_4_^·−^, this result indicates that radical-mediated oxidation contributed substantially to OFL degradation in the Fe(III)/RES/PDS system.^5^^,^^7^^,^^24^ In contrast, the residual OFL concentration remained low in the presence of 0.5–2 mM *tert*-butyl alcohol (TBA), suggesting that ^·^OH made only a minor contribution under the tested conditions. When the TBA concentration was increased to 15 mM, residual OFL increased slightly but remained much lower than that observed with IPA. To further quantify the relative contribution of radical species, the scavenging results were analyzed using competition kinetics based on the second-order rate constants of OFL, IPA, and TBA toward SO_4_^·−^ and ^·^OH (Tables S2–S5). The calculated contributions were 81.9% ± 5.6% for SO_4_^·−^ and 18.9% ± 5.5% for ^·^OH, while non-radical pathways contributed no more than 2% (Tables S2–S5).^28^^,^^29^

**Figure 7:**
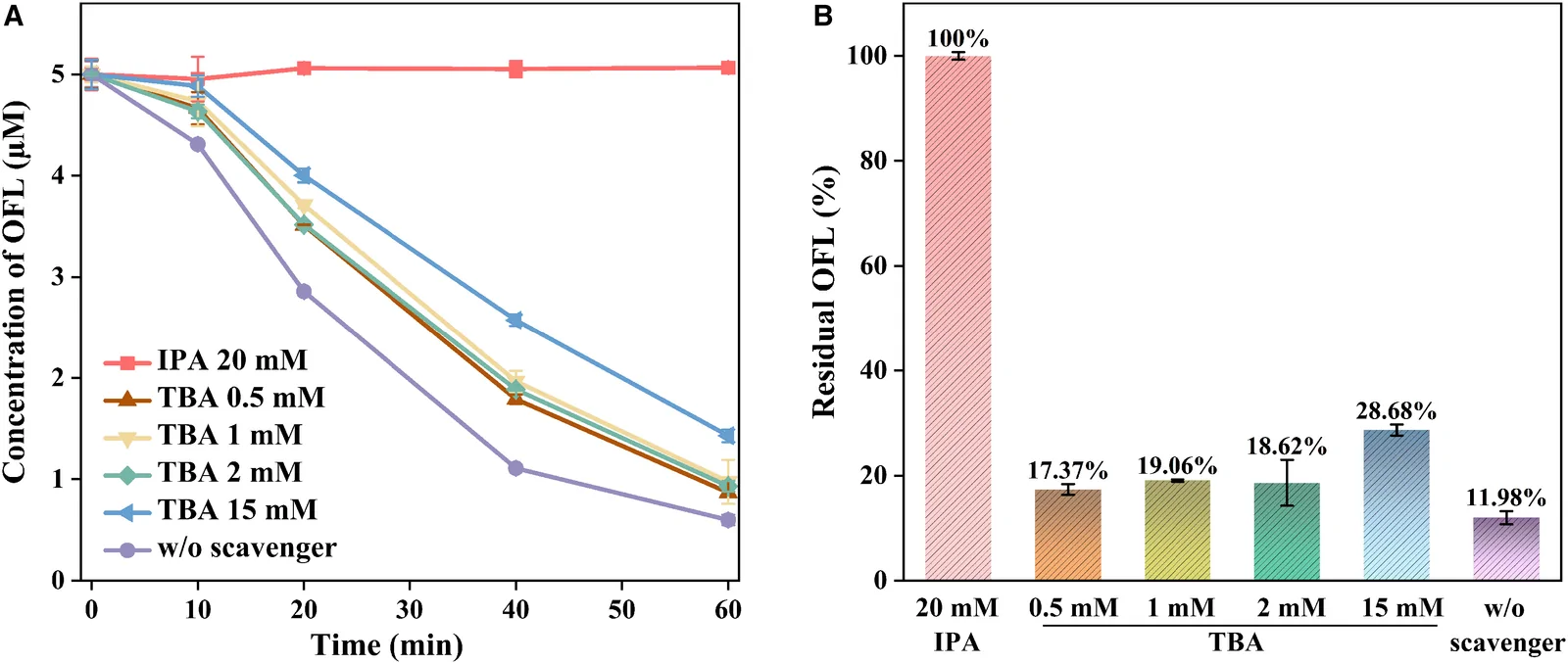
Scavenging experiments in the Fe(III)/RES/PDS system (A) OFL concentration. (B) Residual OFL. All experiments were conducted at [OFL]_0_ = 5 μM, [Fe(III)]_0_ = 100 μM, [PDS]_0_ = 500 μM, [RES]_0_ = 10 μM, and pH = 6.5. Error bars represent mean ± SD (*n* = 3).

### OFL degradation in simulated surface water matrix

The presence of different anions markedly changed the OFL degradation profiles (Figures 8A and 8B). In the ion-free system, 88% of OFL was degraded after 60 min. The addition of Cl^−^ resulted in a comparable degradation efficiency of 84.7% ± 1.1%, while NO_3_^−^ showed no adverse effect, with 90.8% ± 1.3% OFL degradation. In contrast, NO_2_^−^ almost completely suppressed OFL removal, leaving most OFL undegraded after 60 min and giving only 1.3% ± 0.7% degradation. A similar response was observed in the mixed ion system containing Cl^−^, NO_2_^−^, and NO_3_^−^, indicating that NO_2_^−^ was mainly responsible for the poor OFL degradation in the simulated surface water matrix.

**Figure 8:**
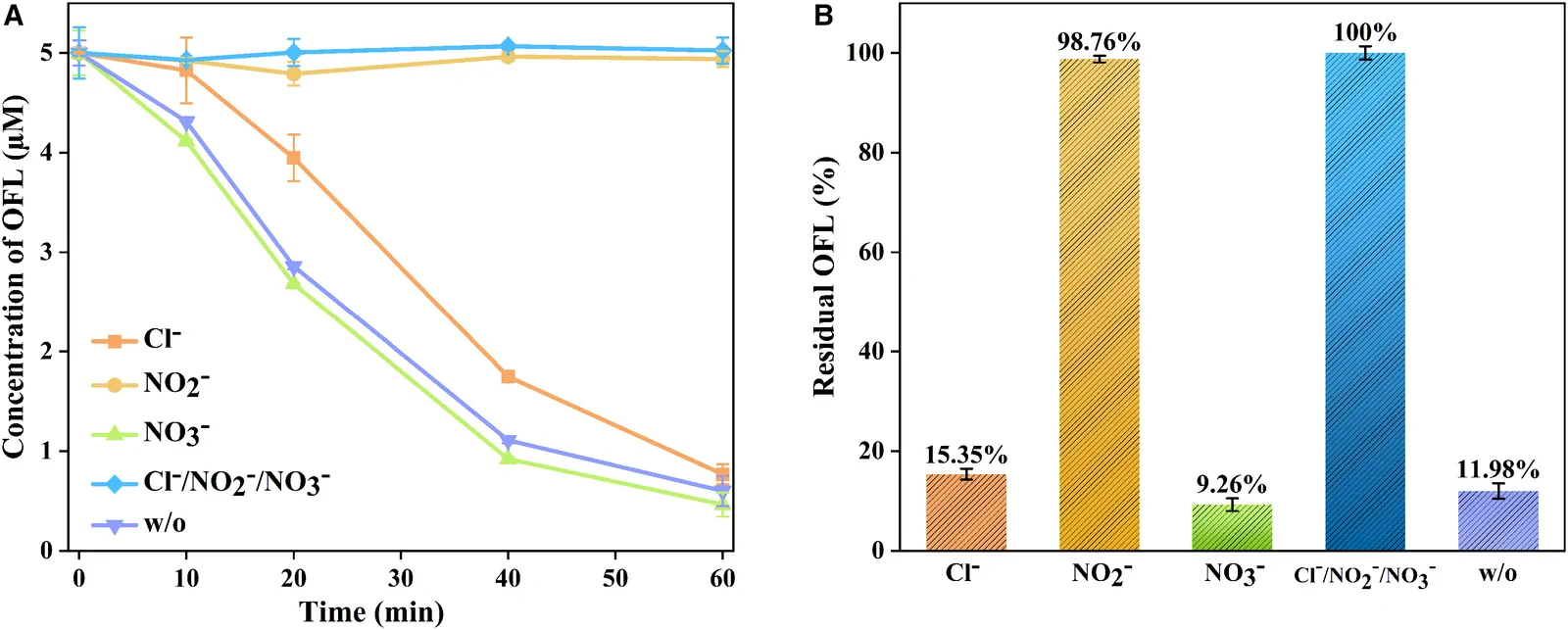
OFL degradation in the presence of inorganic ions (A) OFL concentration. (B) Residual OFL. All experiments were conducted at [OFL]_0_ = 5 μM, [Fe(III)]_0_ = 100 μM, [PDS]_0_ = 500 μM, [RES]_0_ = 10 μM, and pH = 6.5. Error bars represent mean ± SD (*n* = 3).

## Discussion

### Role of RES in iron redox cycling

The Fe(II) quantification results provide the first insight into the role of RES in the Fe(III)/PDS system (Table 1). The formation of Fe(II), which is generally regarded as the more active iron species for initiating PDS activation, indicates that RES can reduce Fe(III) under the tested conditions.^23^^,^^30^ However, Fe(III) is partially reduced (from 5% to 20% according to the tested conditions), thus suggesting that RES should not be regarded as a strong stoichiometric reductant that directly converts most Fe(III) into Fe(II). Instead, its role is more likely associated with maintaining a partial but continuous Fe(III)/Fe(II) redox cycle. This interpretation is important because PDS activation by iron species depends not only on the amount of Fe(II) but also on the regeneration of Fe(II) from Fe(III) during the reaction.^11^^,^^13^^,^^16^

The limited Fe(II) yield may be related to the relatively high Fe(III) molar ratios used in the reaction system and the limited aqueous solubility of RES.^31^ Previous studies using ascorbic acid or protocatechuic acid have shown that small organic reductants or phenolic ligands can enhance iron-based peroxide or persulfate activation through iron complexation, electron transfer, and Fe(III)/Fe(II) cycling.^19^^,^^20^ However, RES differs from these mediators because it has a stilbene backbone, lacks carboxyl groups and adjacent catechol-type hydroxyl groups, and shows relatively low aqueous solubility.^9^^,^^18^^,^^23^ These structural and physicochemical features may restrict its direct reducing ability toward Fe(III), which is consistent with the partial Fe(III) reduction observed in this study. Therefore, the present results do not suggest that RES is superior to previously reported polyphenols but indicate that a structurally different natural polyphenol can still support Fe(III)/Fe(II) redox cycling within an appropriate concentration window. In addition, Fe(III)/Fe(II) redox conversion can be affected by pH, ligand environment, and complexation state, which may influence the measured Fe(II) yield.^32^ Therefore, the role of RES in this system lies not in complete Fe(III) reduction but in limited redox mediation that can provide a partial pathway for Fe(II) regeneration during PDS activation.^30^

### System efficiency and reagent balance

The comparison among different reagent systems further clarifies why RES was introduced into the Fe(III)/PDS system (Figure 2). The limited activity of the single-reagent systems indicates that OFL degradation could not be efficiently initiated by Fe(III), PDS, or RES alone. Although Fe(III) can activate PDS, the binary Fe(III)/PDS system is intrinsically constrained by the slow regeneration of Fe(II) from Fe(III), which limits continuous PDS activation and consequently OFL degradation.^11^^,^^33^^,^^34^ The improved performance of the RES-containing ternary system is therefore most reasonably attributed to the ability of RES to support Fe(III)/Fe(II) redox cycling. This interpretation is consistent with previous studies on natural reductant- or phenolic ligand-assisted iron systems, in which organic mediators promoted Fe(III) reduction, improved iron availability, and facilitated Fe(III)/Fe(II) cycling for enhanced peroxide or persulfate activation.^9^^,^^18^^,^^19^^,^^20^ However, the enhancement remained moderate, suggesting that RES has limited reducing capacity and does not completely reduce Fe(III) under the tested conditions. Thus, this observation supports the role of RES as a redox mediator rather than a strong stoichiometric reducing agent.

The results of reagent optimization indicate that the performance of the RES/Fe(III)/PDS system is governed by the balance among their concentrations. The PDS concentration experiment shows that increasing oxidant concentration can improve OFL degradation only within a certain range (Figure 3). Once sufficient PDS was available, further increasing the oxidant concentration produced limited additional benefit and even had a detrimental effect. This suggests that PDS availability was not the only factor controlling OFL degradation. It also suggests that the OFL degradation efficiency was not only controlled by PDS availability alone but also by the efficiency of PDS activation.

The Fe(III) concentration trend further supports this interpretation (Figure 4). At relatively low Fe(III) concentrations, increasing Fe(III) can provide more reduced iron species and thereby favor PDS activation.^11^^,^^13^^,^^16^ However, once Fe(III) was sufficiently supplied, additional Fe(III) produced only a negligible improvement. This indicates that the limiting factor was no longer the amount of Fe(III) but the efficiency of Fe(III)-to-Fe(II) conversion supported by RES. Such behavior is consistent with iron-based persulfate systems, in which continuous Fe(III)/Fe(II) redox cycling is required to sustain radical generation.^11^^,^^13^^,^^14^ Therefore, using more Fe(III) does not necessarily lead to a proportional increase in degradation efficiency, since it requires RES to generate Fe(II) for efficient PDS activation.

The RES-dependent trend is particularly important because it reflects the concentration-sensitive function of RES in the Fe(III)/PDS system (Figure 5). At moderate concentrations, RES promoted OFL degradation by supporting Fe(III) reduction and facilitating iron redox cycling.^9^^,^^18^^,^^35^ This aligns with the role of polyphenolic compounds as electron-donating ligands and/or redox mediators in iron-based AOPs.^9^^,^^18^ However, the decrease in degradation efficiency at the highest RES concentration indicates that RES can also introduce competing reactions. Indeed, RES is an antioxidant phenolic compound that can react with radical species at elevated concentrations.^13^^,^^24^ Therefore, maintaining an appropriate concentration window in which Fe(III)/Fe(II) redox cycling is enhanced without excessive radical quenching is crucial to sustain the efficiency of the RES/Fe(III)/PDS system. Similar concentration-dependent behavior has been reported for protocatechuic acid-assisted Fe/PDS systems, where an appropriate mediator dose favored Fe cycling, whereas excessive mediator addition hindered Fe-redox circulation and consumed reactive species.^20^

Taken together, the optimization experiments show that the RES/Fe(III)/PDS system requires coordinated control of PDS, Fe(III), and RES concentrations. PDS provides the oxidant source, Fe(III) supplies the iron redox center, and RES assists Fe(III)/Fe(II) redox cycling. Although each component becomes less beneficial once it exceeds a certain concentration range, the main limiting factor is the concentration of RES. An optimal balance between the amounts of all reagents is the key for maintaining effective PDS activation with controlled oxidant, iron, and mediator concentrations.

### Radical contribution to OFL degradation

The competition-kinetic analysis of the scavenging experiments further supports a sulfate radical-dominated oxidation process in the RES/Fe(III)/PDS system. In persulfate-based AOPs, PDS activation commonly produces SO_4_^·−^ and ^·^OH as major reactive radical species, both of which can contribute to the oxidative degradation of organic contaminants.^5^^,^^7^ The nearly complete suppression of OFL degradation by IPA indicates that radical-mediated oxidation was essential in this system, because IPA can efficiently quench both SO_4_^·−^ and ^·^OH.^24^^,^^36^^,^^37^^,^^38^ This result further indicates that radical-mediated oxidation played an important role in OFL removal.

The different response to TBA further helps distinguish the relative contribution of SO_4_^·−^ and ^·^OH. TBA reacts much more rapidly with ^·^OH than with SO_4_^·−^, making it more sensitive to ^·^OH-related inhibition in sulfate radical-based systems.^37^^,^^38^ Therefore, the limited effect of TBA at lower concentrations suggests that ^·^OH was not the dominant contributor under the tested conditions. In contrast, the persistence of OFL degradation in the presence of TBA is more likely driven by SO_4_^·−^.

The contrasting effects of IPA and TBA, together with the competition-kinetic calculation, indicate that SO_4_^·−^ was the predominant radical contributor in the RES/Fe(III)/PDS system, accounting for approximately 82% of OFL degradation, while ^·^OH contributed approximately 19%. This interpretation is also consistent with the favorable performance of the system under circumneutral conditions, since SO_4_^·−^ generally shows broader pH adaptability in SR-AOPs than ^·^OH.^5^^,^^7^^,^^39^

### Practical relevance in water treatment

The pH-dependent behavior reflects the combined influence of RES-mediated redox cycling, iron availability, and OFL speciation in the Fe(III)/PDS system. Although acidic conditions generally help maintain dissolved iron species, excessively low pH did not favor OFL degradation in this study. This is more likely related to the negative effect of strongly acidic conditions on RES, which may affect its interaction with Fe(III) and weaken its role in sustaining the Fe(III)/Fe(II) cycling.^13^^,^^30^^,^^32^

The decreased activity under alkaline conditions may be attributed to iron hydrolysis and precipitation, which can reduce the concentration of dissolved iron species available for PDS activation.^11^^,^^15^ At elevated pH, dissolved iron species are more likely to transform into less available hydrolyzed or precipitated forms, reducing the amount of active iron available for PDS activation.^11^^,^^15^^,^^40^ Changes in pH may also alter OFL speciation because of its ionizable carboxyl and piperazinyl groups (pK_a_ ≈ 6.0 and 8.2), thereby influencing its interaction with reactive radicals.^1^^,^^3^^,^^41^

Based on the best performance observed at pH 6.5, the present study suggests that the RES/Fe(III)/PDS system is practically relevant under circumneutral conditions, where iron availability, RES-mediated redox cycling, and radical utilization may be more favorably balanced. Such circumneutral operation is advantageous for water treatment, as extensive pH adjustment at large scale would require additional chemicals and increase operational costs.

The experiment in simulated surface water matrix highlights the strong dependence of sulfate radical-based AOP on matrix composition (Figure 8). The limited influence of Cl^−^ and NO_3_^−^ suggests that these ions did not act as major radical competitors under the tested conditions. In contrast, NO_2_^−^ can rapidly react with SO_4_^·−^, thereby reducing the availability of SO_4_^·−^ for OFL oxidation.^42^^,^^43^^,^^44^ Therefore, the presence of NO_2_^−^ introduces a competing pathway for radical consumption.^41^ This explains why the mixed ion system was mainly governed by the inhibitory effect of NO_2_^−^ rather than by Cl^−^ and NO_3_^−^. More broadly, these results indicate that the performance of the RES/Fe(III)/PDS system cannot be evaluated only in pure water, because specific wastewater components may substantially alter radical utilization and oxidation efficiency.^24^^,^^45^

Overall, this study highlights the potential of natural polyphenols as redox mediators for improving Fe-based SR-AOPs under mild reaction conditions. Although it is an antioxidant, RES provides a molecular strategy for regulating iron redox cycling and improving the utilization of PDS in circumneutral water treatment. The results also show that the effectiveness of such systems should not be judged only by pollutant degradation efficiency but by the balance among redox mediation, radical generation, and matrix tolerance. This perspective may support the broader development of natural antioxidant-assisted persulfate-based AOPs for the treatment of antibiotic-contaminated waters and other complex aqueous matrices.

### Limitations of the study

This study provides preliminary evidence for the role of RES in Fe(III)/PDS system and OFL degradation, while several aspects remain open for further investigation. The degradation intermediates, transformation pathways, and toxicity evolution of OFL were not examined in detail, and future analysis would help better assess the environmental outcome of such AOPs. Chemical scavenging combined with competition-kinetic analysis supported the predominant contribution of SO_4_^·−^, while direct detection of short-lived reactive species would provide further mechanistic resolution. Fe(II) formation supports the redox role of RES, whereas direct spectroscopic evidence for RES-Fe(III) coordination would further clarify the initial interaction between RES and iron species. In addition, the simulated surface water matrix focused on selected inorganic ions, whereas real water matrices contain more constituents that may influence iron cycling and radical utilization. These aspects should be addressed in future studies to enable a more comprehensive evaluation of the RES/Fe(III)/PDS system.

## Resource availability

### Lead contact

Requests for further information and resources should be directed to and will be fulfilled by the lead contact, Martin Motola (martin.motola@uniba.sk).

### Materials availability

This study did not generate new unique reagents or materials.

### Data and code availability

• All data reported in this paper will be shared by the lead contact upon request.
• This paper does not report original code.
• Any additional information required to reanalyze the data reported in this paper is available from the lead contact upon request.

## Acknowledgments

This work was supported by the Shanghai Sci-tech Co-research Program funded by Science and Technology Commission of Shanghai Municipality (no. 25HB2711800), the Chinese Scholarship Council (no. 202508310228), the Project of Ministry of Science and Technology of China for China-Slovakia international cooperation (no. 9-7), Science and Technology Development Fund of Pudong New Area (PKJ2024-N03), and the Scientific Grant Agency of the Slovak Ministry of Education, Sciences, Research and Sport (VEGA) # 1/0319/23.

## Author contributions

Conceptualization, Y.Z., O.M., and M.M.; data curation, W.Y., Y.W., and Y.Z.; formal analysis, Y.Z.; investigation, W.Y., Y.W., D.H., and W.D.; methodology, W.Y., Y.W., Y.Z., D.H., and W.D.; visualization, Y.Z.; writing – original draft, Y.Z. and W.Y.; writing – review & editing, Y.Z., O.M., and M.M.; supervision, O.M. and M.M.; project administration, O.M. and M.M.; funding acquisition, O.M. and M.M.

## Declaration of interests

The authors declare no competing interests.

## STAR★Methods

### Key resources table

**Table:** 

REAGENT or RESOURCE	SOURCE	IDENTIFIER
**Chemicals**		

Ofloxacin, OFL, 99%	Aladdin	CAS: 82419-36-1
Resveratrol, RES, 99%	Aladdin	CAS: 501-36-0
Sodium persulfate, Na2S2O8, ≥98%	Aladdin	CAS: 7775-27-1
Iron(III) perchlorate, Fe(ClO4)3, 98%	Aladdin	CAS: 13537-24-1
Sodium acetate, CH3COONa, 97%	Aladdin	CAS: 127-09-3
Acetic acid, CH3COOH, ≥99.5%	Aladdin	CAS: 64-19-7
1,10-Phenanthroline, ≥99%	Aladdin	CAS: 66-71-7
Hydroxylamine hydrochloride, HONH_2_·HCl, 99%	Aladdin	CAS: 5470-11-1
Isopropanol, 2-Pr, ≥99.7%	Aladdin	CAS: 67-63-0
tert-Butanol, t-BuOH, ≥98%	Aladdin	CAS: 75-65-0
Sodium nitrite, NaNO2	Aladdin	CAS: 7632-00-0
Sodium nitrate, NaNO3	Aladdin	CAS: 7631-99-4
Sodium chloride, NaCl	Aladdin	CAS: 7647-14-5
Methanol, CH3OH, HPLC grade	Aladdin	CAS: 67-56-1
Acetonitrile, 99%	Aladdin	CAS: 75-05-8
Phosphoric acid, H3PO4	Aladdin	CAS: 7664-38-2
Perchloric acid, HClO4, ≥99.5%	Aladdin	CAS: 7601-90-3
Sodium hydroxide, NaOH, ≥99%	Aladdin	CAS: 1310-73-2

**Software and algorithms**		

Origin 2024 software	OriginLab Corporation	Version 10.1.0.178

**Other**		

High-performance liquid chromatography (HPLC)	Dionex, USA	UltiMate3000

### Method details

#### Materials and reagents

Key reagents, materials, software, and instruments used in this study are listed in the key resources table. Unless otherwise specified, all chemicals were used as received without further purification. All aqueous solutions were prepared with ultrapure water with a resistivity of 18.2 MΩ cm.

#### Fe(II) quantification

The redox interaction between RES and Fe(III) was examined in the absence of PDS to evaluate the genuine RES redox properties. The generated Fe(II) was quantified using the 1,10 phenanthroline spectrophotometric method.^25^^,^^34^ Briefly, Fe(II) forms a stable orange red complex with 1,10 phenanthroline in an acetic acid and sodium acetate buffer solution at approximately pH 5.5. The complex shows a characteristic absorbance at 510 nm,^34^^,^^35^ from which the Fe(II) concentration was calculated based on the corresponding calibration curve.^26^

#### OFL degradation experiments

OFL degradation experiments were conducted to evaluate the performance of Fe(III)-based PDS activation in the presence of RES. All degradation experiments were performed at 25 ± 1 °C in 100 mL brown light protective glass bottles, with a total reaction volume of 50 mL. Magnetic stirring was maintained throughout the reaction to ensure solution homogeneity. Single reagent, binary, and ternary systems were prepared using different combinations of Fe(III), RES, and PDS. At predetermined time intervals, 1.0 mL of reaction solution was collected by microfilters, and immediately mixed with 0.1 mL of methanol to quench the reaction.

#### HPLC analysis of OFL

The residual concentration of OFL was determined by HPLC equipped with C18 column (5 μm, 4.6 mm × 150 mm) maintained at 40 °C. The mobile phase consisted of acetonitrile and an aqueous solution containing 0.1% phosphoric acid (pH ≈ 3.0) at a volume ratio of 30:70. Isocratic elution was performed at a flow rate of 1.0 mL/min, with an injection volume of 20 μL and a detection wavelength of 295 nm. Under these chromatographic conditions, OFL was detected at a retention time of approximately 4.8 min.

#### Optimization of reaction parameters

The reaction parameters were optimized sequentially in the Fe(III)/RES/PDS system. The PDS concentration was first investigated by varying the initial PDS concentration at 100, 250, 500, and 1000 μM, while maintaining [Fe(III)]_0_ = 100 μM and [RES]_0_ = 10 μM. Based on the PDS optimization results, 500 μM PDS was selected for the following experiments.

The Fe(III) concentration was then examined at initial Fe(III) concentrations of 50, 100, 150, 200, and 300 μM, while maintaining [PDS]_0_ = 500 μM and [RES]_0_ = 10 μM. Based on this experiment, 150 μM Fe(III) was selected for subsequent optimization.

The RES concentration was further evaluated at initial RES concentrations of 5, 7.5, 10, 15, and 20 μM, while maintaining [PDS]_0_ = 500 μM and [Fe(III)]_0_ = 150 μM. Finally, the initial pH was investigated over a pH range of 2.0, 3.5, 5.0, 6.5, 7.5, 9.0, and 10.5, while maintaining the initial concentrations of OFL, Fe(III), PDS, and RES at 5, 150, 500, and 10 μM, respectively.

#### Scavenging experiments

Chemical scavenging experiments were conducted to assess the contribution of radical species involved in OFL degradation in the Fe(III)/RES/PDS system. *Tert*-butyl alcohol (TBA) and isopropanol (IPA) were selected as scavengers because of their different reaction rates with ^·^OH and SO_4_^·-^.^38^ TBA reacts rapidly with ^·^OH, with a reported rate constant of k_·OH, TBA_ = 4.2 × 10^8^ M^−1^ s^−1^, whereas its reaction with SO_4_^·-^ is much slower, with a rate constant of k_SO4·-, TBA_ = 9.1 × 10^5^ M^−1^ s^−1^.^37^^,^^38^ In contrast, IPA reacts efficiently with both ^·^OH and SO_4_^·-^, with rate constants of k_·OH, IPA_ = 1.9 × 10^9^ M^−1^ s^−1^ and k_SO4·-, IPA_ = (1.2–8.5) × 10^7^ M^−1^ s^−1^.^22^^,^^34^^,^^35^^,^^36^

Based on previous reports, OFL reacts rapidly with both SO_4_^·-^ and ^·^OH, with rate constants of k_SO4·-, OFL_ = 2.19 × 10^10^ M^−1^ s^−1^; k_·OH, OFL_ = 3.97 × 10^9^ M^−1^ s^−1^.^39^ Based on these reactivities, the scavenger concentrations were selected. IPA was added at 20 mM as a broad radical scavenger for both ^·^OH and SO_4_^·-^, while TBA was added at 0.5, 1, 2, and 15 mM to distinguish the role of ^·^OH and to evaluate radical competition at elevated scavenger concentrations.

#### Simulated surface water treatment

To evaluate the applicability of the Fe(III)/RES/PDS system in simulated surface water matrix, OFL degradation was examined in a simulated surface water matrix solution based on the reported water quality of Shajing Harbor, Shanghai, China. According to these data, the average concentrations of common ions, including Cl^−^, NO_2_^−^, NO_3_^−^, and SO_4_^2−^, in the surface water were 51.50, 1.80, 9.97, and 68.23 mg/L, respectively. These values correspond to 1.45 mM Cl^−^, 40 μM NO_2_^−^, 160 μM NO_3_^−^, and 0.71 mM SO_4_^2−^.

Since SO_4_^2−^ is already the stable end form of sulfate species and is not expected to be converted into SO_4_^·-^ or to participate significantly in the Fe(III)/RES/PDS reaction, the matrix experiments focused on Cl^−^, NO_2_^−^, and NO_3_^−^. NaCl, NaNO_2_, and NaNO_3_, which were added individually or simultaneously to the Fe(III)/RES/PDS system under the following reaction conditions: 100 μM Fe(III), 10 μM RES, 500 μM PDS, and pH 6.5. Because NO_2_^−^ has been reported to react rapidly with SO_4_^·-^, with a rate constant of k K _SO4·_-, _NO2-_ = 8.8 × 10^8^ M^−1^ s^−1^, particular attention was paid to its possible competition with OFL for reactive species.^40^^,^^41^ Considering that OFL also reacts rapidly with SO_4_^·-^ and ^·^OH,^39^ the presence of NO_2_^−^ may strongly affect radical utilization during OFL degradation.^22^^,^^42^

### Quantification and statistical analysis

The degradation efficiency of OFL was calculated using the following equation:

$$\text{Degradation}\;\text{efficiency}\;\left(\%\right)=\left(1-C_{t}/C_{0}\right)\times 100$$

where *C*_*0*_ is the initial OFL concentration and *C*_*t*_ is the OFL concentration at reaction time *t*. The apparent pseudo first order rate constant was obtained from the linear fitting of ln(*C*_*0*_/*C*_*t*_) versus reaction time.

Unless otherwise specified, degradation experiments were independently repeated three times (*n* = 3), and data are presented as mean ± SD. Data processing and fitting were performed using software listed in the key resources table. No statistical hypothesis testing was performed unless otherwise stated, and therefore no tests of statistical assumptions were applied.
