# Supplementary material for: Resveratrol-mediated Fe(III)/peroxydisulfate activation for ofloxacin degradation at circumneutral pH
Source: iScience. 2026 Sep 22;29(10):117733. doi: 10.1016/j.isci.2026.117733 (PMC13671365; doi:10.1016/j.isci.2026.117733)
Supplement: Supplementary file 1 [file mmc1.pdf]

iScience, Volume *29*

Supplemental information

Resveratrol-mediated Fe(III)/peroxydisulfate activation for ofloxacin degradation at circumneutral pH

Yanlin Wu (吴彦霖), Wenyu Yang (杨文语), Yilan Zeng (曾一览), Dingfeng Huang (黄鼎峰), Wenbo Dong (董文博), Olivier Monfort, and Martin Motola

Supporting Information

This PDF file includes:

Supplementary Table S1-S5

Supplementary Method S1. Competition kinetic calculations for scavenger selection and estimation of radical contributions

Supplementary References

Supplementary Tables

**Scavenger**	k_{obs} **(10^{-3} min^{-1})**	R^2
IPA 20 mM	-0.3 ± 0.2	-
TBA 0.5 mM	33.8 ± 0.9	0.99
TBA 1 mM	31.9 ± 1.3	0.99
TBA 2 mM	32.2 ± 1.0	0.99
TBA 15 mM	24.5 ± 1.5	0.99
w/o	40.2 ± 2.6	0.99

Supplementary Table S1. First-order rate constants (k_{obs}) of quenching experiment (Figure 7).

Reaction	k (M^{-1} s^{-1})	Ref.
$^{\bullet}OH$ + OFL	3.97×10^{9}	[1]
$SO_4^{\bullet-}$ + OFL	2.19×10^{10}	[1]
$^{\bullet}OH$ + TBA	6.0×10^{8}	[2]
$SO_4^{\bullet-}$ + TBA	9.1×10^{5}	[3]
$^{\bullet}OH$ + IPA	1.9×10^{9}	[2]
$SO_4^{\bullet-}$ + IPA	8.5×10^{7}	[4]

Supplementary Table S2. Second-order rate constants for TBA and IPA.

**Conc.**	$k_{TBA,{}^{\bullet}OH}\cdot$[TBA] (s^{-1})	$k_{TBA,SO_4^{\bullet-}}\cdot$[TBA] (s^{-1})	$k_{TBA,{}^{\bullet}OH}\cdot$[TBA]/$k_{OH^{\bullet},OFL}\cdot$[OFL]$_0$	$k_{TBA,{}^{\bullet}SO_4^{-}}$[TBA]/$k_{SO_4^{\bullet-},OFL}\cdot$[OFL]$_0$
0.5 mM	3.00×10^5	4.55×10^2	15.1	0.004
1 mM	6.00×10^5	9.10×10^2	30.2	0.008
2 mM	1.20×10^6	1.82×10^3	60.5	0.017
15 mM	9.00×10^6	1.37×10^4	453	0.125

Supplementary Table S3. Competition analysis of the TBA doses.

**Conc.**	$k_{IPA,HO\bullet}\cdot$[IPA] (s^{-1})	$k_{IPA,SO_4^{\bullet-}}\cdot$[IPA] (s^{-1})	$k_{IPA,{}^{\bullet}OH}\cdot$[IPA]/$k_{OH^{\bullet},OFL}\cdot$[OFL]$_0$	$k_{IPA,{}^{\bullet}SO_4^{-}}$[IPA]/$k_{SO_4^{\bullet-},OFL}\cdot$[OFL]$_0$
20 mM	3.80×10^7	1.70×10^6	1914	15.5

Supplementary Table S4. Competition analysis of the IPA doses.

**Oxidant**	**Contribution (%)**
$SO_4^{\bullet-}$	81.9 ± 5.6
$^{\bullet}OH$	18.9 ± 5.5
Non-radical pathways	≤ 2 *

Supplementary Table S5. Contribution of radicals to OFL degradation. *The value for non-radical pathways is an upper bound set by the analytical uncertainty, since 20 mM IPA suppressed OFL removal completely.

Supplementary Methods S1. Competition kinetic calculations for scavenger selection and estimation of radical contributions, related to Figure 7

1. Rationale for scavenger concentrations

To move beyond a qualitative assignment, the scavenging experiments were designed and interpreted on the basis of competition kinetics. The relevant second-order rate constants are summarized in Table S2. Because TBA reacts with $^{\bullet}OH$ about 660 times faster than with $SO_4^{\bullet-}$, whereas IPA reacts rapidly with both, the two alcohols can be used to partition the oxidant pool, provided their doses are chosen so that each competes with OFL to the intended extent.

With $[OFL]_0 = 5.0\ \mu M = 5.0 \times 10^{-6}$ M:

$k(^{\bullet}OH, OFL)\cdot[OFL]_0 = 3.97 \times 10^9 \times 5.0 \times 10^{-6} = 1.99 \times 10^4\ s^{-1}$

$k(SO_4^{\bullet-}, OFL)\cdot[OFL]_0 = 2.19 \times 10^{10} \times 5.0 \times 10^{-6} = 1.10 \times 10^5\ s^{-1}$

A ratio higher than 10 was used as the criterion for effective scavenging, indicating that more than 90% of the corresponding reactive species is consumed by the quencher rather than by OFL (Eq. S1). Conversely, a ratio below 0.1 indicates that the species is left essentially undisturbed, less than 10% of it being diverted to the quencher (Eq. S2).

For TBA to act as a selective $^{\bullet}OH$ probe, both conditions must be satisfied simultaneously: Eq. S1 applied to $^{\bullet}OH$ and Eq. S2 applied to $SO_4^{\bullet-}$. For IPA, which is intended to remove both radicals, Eq. S1 must be satisfied for each of them.

$$\frac{C_{quencher} * k_{quencher,\bullet OH}}{C_{OFL} * k_{OFL,\bullet OH}} > 10 \qquad (S1)$$

$$\frac{C_{quencher} * k_{quencher,SO4\bullet-}}{C_{OFL} * k_{OFL,HSO4\bullet-}} < 0.1 \qquad (S2)$$

Applying Eq. S1 to $^{\bullet}OH$: [TBA] > 10 × 1.99 × 10^4 / 6.0 × 10^8 = 3.3 × 10^{-4} M = 0.33 mM. Applying Eq. S2 to $SO_4^{\bullet-}$: [TBA] < 0.1 × 1.10 × 10^5 / 9.1 × 10^5 = 1.20 × 10^{-2} M = 12.0 mM. TBA is therefore selective only between 0.33 and 12.0 mM. We tested 0.5, 1 and 2 mM, all inside this window. These doses capture 93.8%, 96.8% and 98.4% of $^{\bullet}OH$, but remove only 0.4%, 0.8% and 1.6% of $SO_4^{\bullet-}$ (Table S3). We also tested 15 mM as a control above the upper limit.

For [IPA] > 10 × 1.10 × 10^5 / 8.5 × 10^7 = 1.29 × 10^{-2} M = 12.9 mM ($SO_4^{\bullet-}$ is binding; the •OH criterion is met at only 0.10 mM). A dose of 20 mM was used, which competitively consumed 94.0% of $SO_4^{\bullet-}$ and more than 99.9% of $^{\bullet}OH$ (Table S4).

2. Contributions of radicals

Contributions of radicals were calculated from the rate constants based on Eqs S3-S5:

$$(\bullet\ \mathrm{OH})\% = \frac{k_{obs} - k_{obs}TBA}{k_{obs0}} \qquad (S3)$$

$$(\mathrm{SO4}\bullet-)\% = \frac{k_{obs}TBA - k_{obs}IPA}{k_{obs0}} \qquad (S4)$$

$$(\mathrm{Non-radical})\% = \frac{k_{obs}IPA}{k_{obs0}} \qquad (S5)$$

Here k_{obs}TBA represents the mean K_{obs} value of 0.5, 1 and 2 mM TBA from Table S1 (32.6 ± 0.6) ×10^{-3} min^{-1}, which are statistically indistinguishable and therefore show that the $^{\bullet}OH$ was already fully quenched at the lowest dose. The 15 mM TBA was excluded because it lies outside the selective window.

References

[1] X. H. Liu, L. Ying, S.Y. Lu, Z. Wang, Y. Q. Wang, G. D. Zhang, X. C. Guo, W. Guo, T. T. Zhang, and B. D. Xi. Degradation difference of ofloxacin and levofloxacin by UV/H_2O_2 and UV/PS (persulfate): Efficiency, factors and mechanism[J]. Chemical Engineering Journal, 2020, 385: 123987.

[2] G. V. Buxton, C. L. Greenstock, W. P. Helman, A. B. Ross. Critical Review of rate constants for reactions of hydrated electrons, hydrogen atoms and hydroxyl radicals ($^{\bullet}OH$ /$^{\bullet}O^-$) in Aqueous Solution[J]. Journal of physical and chemical reference data, 1988, 17(2): 513-886

[3] E. Hayon, A. Treinin, J. Wilf. Electronic spectra, photochemistry, and autoxidation mechanism of the sulfite-bisulfite-pyrosulfite systems. the SO_2^-, SO_3^-, SO_4^-, and SO_5^- radicals. Journal of the American Chemical Society, 1972, 94(1), 47-57.

[4] L. Dogliotti, E. Hayon. Flash Photolysis of Persulfate Ions in Aqueous Solutions. Study of the Sulfate and Ozonide Radical Anions [J]. The Journal of Physical Chemistry, 1967, 71(8): 2511-2516.
